# Differentiation in Neuroblastoma: Diffusion-Limited Hypoxia Induces Neuro-Endocrine Secretory Protein 55 and Other Markers of a Chromaffin Phenotype

**DOI:** 10.1371/journal.pone.0012825

**Published:** 2010-09-17

**Authors:** Fredrik Hedborg, Reiner Fischer-Colbrie, Nurtena Östlin, Bengt Sandstedt, Maxine G. B. Tran, Patrick H. Maxwell

**Affiliations:** 1 Rudbeck Laboratory, Department of Genetics and Pathology, Uppsala University, Uppsala, Sweden; 2 Department of Womens' and Children's Health, Uppsala University, Uppsala, Sweden; 3 Department of Pharmacology, Innsbruck Medical University, Innsbruck, Austria; 4 Childhood Cancer Research Unit, Karolinska Institutet, Stockholm, Sweden; 5 Cancer Research UK, Cancer Research Institute, Li Ka Shing Centre, Cambridge University, Cambridge, United Kingdom; 6 Division of Medicine, University College London, London, United Kingdom; Brigham and Women's Hospital, United States of America

## Abstract

**Background:**

Neuroblastoma is a childhood malignancy of sympathetic embryonal origin. A high potential for differentiation is a hallmark of neuroblastoma cells. We have previously presented data to suggest that *in situ* differentiation in tumors frequently proceeds along the chromaffin lineage and that decreased oxygen (hypoxia) plays a role in this. Here we explore the utility of Neuro-Endocrine Secretory Protein 55 (NESP55), a novel member of the chromogranin family, as a marker for this process.

**Methodology/Principal Findings:**

Immunohistochemical analyses and *in situ* hybridizations were performed on human fetal tissues, mouse xenografts of human neuroblastoma cell lines, and on specimens of human neuroblastoma/ganglioneuroma. Effects of anaerobic exposure on gene expression by cultured neuroblastoma cells was analyzed with quantitative real-time PCR. Fetal sympathetic nervous system expression of NESP55 was shown to be specific for chromaffin cell types. In experimental and clinical neuroblastoma NESP55 immunoreactivity was specific for regions of chronic hypoxia. NESP55 expression also correlated strikingly with morphological evidence of differentiation and with other chromaffin-specific patterns of gene expression, including *IGF2* and *HIF2α.* Anaerobic culture of five neuroblastoma cell lines resulted in an 18.9-fold mean up-regulation of *NESP55*.

**Conclusions/Significance:**

The data confirms that chronic tumor hypoxia is a key microenvironmental factor for neuroblastoma cell differentiation, causing induction of chromaffin features and NESP55 provides a reliable marker for this neuronal to neuroendocrine transition. The hypoxia-induced phenotype is the predominant form of differentiation in stroma-poor tumors, while in stroma-rich tumors the chromaffin phenotype coexists with ganglion cell-like differentiation. The findings provide new insights into the biological diversity which is a striking feature of this group of tumors.

## Introduction

“Neuroblastoma, a genetically and clinically diverse malignancy of infancy and early childhood, is derived from sympathetic precursor cells within sympathetic ganglia or the adrenal medulla [1].” Neuroblastoma cells have a remarkably retained capacity for sympathetic differentiation, both on the basis of histological analysis and clinically through well-documented complete remissions due to spontaneous maturation of residual tumor tissue. The molecular mechanisms underlying such differentiation are not clearly understood, but *in vitro* similar changes are induced by retinoic acid analogues [2], phorbol esters [3] or Schwann cell co-culture [4] and the neurotrophin pathway is considered important [5]. In addition to the accepted notion that neuroblastoma differentiates towards a sympathetic ganglion cell phenotype in vivo [6] and in vitro [7], recent evidence has suggested a neuroendocrine maturation pathway with acquisition of chromaffin features. We previously showed that certain characteristics of sympathetic neuroendocrine cells [8] are frequent in neuroblastoma [9], [10] Interestingly these occur centrally within tumor nodules and in perinecrotic areas, representing regions of chronic tumor hypoxia and *in vitro* these neuroendocrine features are highly induced by hypoxia [11], [12].

Diffusion-limited chronic hypoxia is very common in solid tumors. In neoplastic tissues increased cell proliferation frequently outpaces angiogenesis, resulting in tumor cells extending beyond the oxygenation zones that surround blood vessels. This is exacerbated by the high metabolic demands of tumor cells, and unreliable vessel perfusion [13], [14]. Reduced oxygenation elicits activation of Hypoxia-Inducible Factors (HIFs), representing an evolutionarily conserved physiological response which coordinates a range of adaptive changes in gene expression and cross-communicates with other signaling pathways [13], [15], [16]. The consequences of tumor hypoxia are increasingly recognized as an important aspect of cancer biology. Among other effects, hypoxia promotes resistance to chemotherapeutic agents and radiotherapy, increases angiogenesis, and can drive tumor progression and metastasis [17]–[21]. Its role in altering cell differentiation programmes has received limited attention, and is likely to be dependent on cell context.

In our previous characterization of the hypoxic phenotype in neuroblastoma we employed two markers, *IGF2* and chromogranin A [9], [12], [22]. Within the fetal sympathetic nervous system *IGF2* expression is specific and sensitive for chromaffin cell types [8], but lacks an obvious link to chromaffin function. In neuroblastoma *IGF2* expression is an excellent marker for hypoxia, with more than 100-fold induction reported [12]. Chromogranin A is a major constituent of neurosecretory granules and is therefore an established marker for neuroendocrine cell function [23], [24]. In clinical neuroblastoma chromogranin A immunoreactivity is associated with tumor hypoxia, but with less dynamic range than *IGF2* expression. In evaluating the role of hypoxia in neuroblastoma, and the extent to which it results in acquisition of a neuroendocrine phenotype, we and others [7] consider identifying additional reliable markers to be essential.

Recently, Neuro-Endocrine Secretory Protein 55 (NESP55), a novel member of the chromogranin family [25], [26], was found to be co-expressed with chromogranin A in neuroblastoma [27]. The aim of this study was to explore the chromaffin marker qualities of NESP55 during human development and whether its presence in neuroblastoma may be a consequence of tumor hypoxia. Our data shows that NESP55 is a highly specific marker for chromaffin cell types during development and strongly support our hypothesis that its expression in neuroblastoma is a function of tumor hypoxia.

## Materials and Methods

### Ethics statement

Animal experiments were approved by the regional animal ethics committee (Uppsala's Ethical Committee on Animal Experiments; approval C70/7), and all testing of clinical tumors and fetal tissues was approved by the regional human ethics committee (Regional Ethical Review Board *in* Uppsala; approval 2007/070). Written informed consent was obtained from families of surviving children.

### Cell culture

The human neuroblastoma cell lines Kelly [28], SK-N-BE(2) (ATCC number CRL-2271), SH-SY5Y (ATCC number CRL-2266), SK-N-FI (ATCC number CRL-2142), and LAN-5 [28], and the human medulloblastoma D324 Med [29] and supratentorial PNET PFSK1 (ATCC number CRL-2060) cell lines were cultured in Eagles minimal essential or RPMI medium supplemented with 10% fetal calf serum, glutamine and antibiotics. For hypoxia, cells at 30% cell density were incubated for 40 hours in sealed chambers designed for anaerobic bacterial culture (GasPac™ system, BD Biosciences, San Jose, CA, USA). 35 ml medium was added to the 10 cm diameter culture dishes prior to incubation. Anaerobic conditions were reached in the gas phase after approximately three hours. Cells were viable following this exposure, as reported previously [12].

### Real-time PCR analyses

Total RNA was prepared with TRIZOL®Reagent (invitrogen™, Sweden). RNA purity was controlled with the 2100 Bioanalyzer™ system (Agilent Technologies, Inc., Santa Clara, CA, USA). After treatment with amplification grade DNase I (invitrogen™), cDNAs were produced using Oligo dT primers (invitrogen™, Sweden), M-MuLV RT and recommended buffers (New England Biolabs Inc, USA). Real-time PCR was performed with SYBR Green (Applied Biosystems, Applera Sweden). Samples were run at 95°C for 10 minutes followed by 45 cycles of 95°C, 15 seconds and 60°C, one minute. The following primers were used : *NESP55:* forward AAGCCCGAGGACAAAGATCC, reverse TTTGGCTTGCAGCGACG, *IGF2*: forward TTCCGGACAACTTCCCCAG, reverse TGGACTGCTTCCAGGTGTCA, *HIF1α*: forward TTGGAACATTATTACAGCAGCCAG, reverse CACTAGATTTGCATCCTTTTACACG, *HIF2α*: forward AGGACTACAGCCTGTCGTCAGC, reverse AAATGAGGGCCCGAGCA, *NOTCH1*: forward CACGGATCATATGGACCGC, reverse CCAGGTTGTACTCGTCCAGCA, *CHROMOGRANIN A*: forward GGTGGCAGGCAAAGAGAGAA, reverse ACTCTCGCCTTTCCGGATCT, *HES1*: forward GGCGGCTAAGGTGTTTGGA, reverse AAGGCCCCGTTGGGAAT, *ID2*: forward ATCCTGCAGCACGTCATCG, reverse GACAATAGTGGGATCCGAGTCC, *β-ACTIN*: forward ATGGATGATGATATCGCCGC, reverse AAGCCGGCCTTGCACAT, *RPS28*: forward ACCGGTTCTCAGGGACAGTG, reverse GATGATGGATCGGCTCGTGT. PCR products were checked for expected size. Results are the mean of two triplicate measurements. To identify an appropriate mRNA species for normalization of values we performed *RPS28* and *β-actin* rt-qPCR of normoxic and anaerobic samples from all cell lines. Variations in *RPS28* expression between the two conditions were insignificant in all cell lines, whereas the *β-actin* analyses indicated robust hypoxic down-regulation. Therefore triplicate measurement of *RPS28* mRNA was chosen for normalization of each sample.

### Experimental tumors

Subcutaneous xenograft tumors were generated in immunodeficient NMRINU-M mice (Taconic Europe) by injecting 7–10 week old female animals with 20–50 million cells in each hind leg. After reaching a maximum size of 1.5 ml, tumors were perfusion fixed and processed to 3 µm serial sections as described [12].

### Clinical tumors

Serial 3–5 µm sections were obtained from routinely processed surgical specimens of 24 neuroblastomas and 2 ganglioneuromas. INSS criteria [30] were used for clinical staging. 21 neuroblastoma specimens showed stroma-poor histology and 3 mixed histology with stroma-poor and stroma-rich regions. The samples represented a wide clinical spectrum, with particular focus on high-risk disease. Serial sections of all tumors were evaluated immunohistochemically for presence of *NESP55*, chromogranin A and with *in situ* hybridization for expression of *VEGFA* and *IGF2*. Analyses were repeated if the initial result was negative. *NESP55 in situ* hybridization was performed on eleven tumors.

### Control tumor specimens

Serial sections were produced from the following pediatric tumors of nervous system origin: 13 gliomas (high and low grade), 9 medulloblastomas, 5 supratentorial primitive neuroectodermal tumors, 4 ependymomas, 2 oligodendrogliomas and 2 gangliogliomas.

### Human fetal specimens

Serial 3 µm sections were produced from six specimens of formalin fixed/paraffin embedded human fetal tissues containing retroperitoneal sympathetic structures. Developmental ages were: 6, 9, 12 (two specimens), 18 weeks and term.

### *In situ* hybridization

^35^S-αUTP (GE Health Care, Uppsala, Sweden)-labeled probes were generated with specific activity of ∼250 Ci/mole probe. Templates were: *NESP55*: a 874 bp Pst1/Bam H1 cDNA fragment of the hGAI34 clone [31]. *HIF1α*: a Bgl2/EcoR1 cDNA fragment spanning amino acids 28 to 329 of the coding region, which generated a 255 bp template at linearization with Sal1. *HIF2α*: a 220 bp EcoR1/Bam H1 cDNA fragment (nucleotides 2542–2762 of sequence accession no. U81984). *Chromogranin A*: a 240 bp H9/P1 cDNA fragment of the human gene (gift from Stefan Wennström, Örebro University, Sweden). *IGF*2, *VEGFA165*, *GAP43*, *β*-*actin* antisense, and *IGF2* sense probes were generated as described [12]. Riboprobes, at 10–80.000 CPM/µl were hybridized to sections overnight at 56°C and washed under hybridization-stringent conditions before RNase treatment, as described [32]. Photographic emulsion (NTBII; Eastman Kodak Co., Rochester), diluted 1∶1 in 2% glycerol, was applied and slides were developed after 1–3 months exposure.

### Immunohistochemical analyses

Antigens were detected as follows: NESP55: antisera were raised in rabbits immunized with a synthetic C-terminal fragment of bovine NESP55 (GAIPIRRH) coupled to keyhole limpet haemocyanin, and affinity purified using the GAIPIRRH epitope. The specificity of the NESP55 antibodies had first been tested on human brain tissue with epitope-adsorbed antibodies, which was repeated on the fetal specimens in this study using adsorbed and non-adsorbed antibodies from the same batch at a four-fold higher concentration than used for all other NESP55 immunohistochemical analyses (1∶250 vs 1∶1000 dilution). The specificity of the affinity-purified anti NESP55 antibodies was also tested on brain sections from *nesp55* knock-out mice (kindly provided by Gavin Kelsey). Chromogranin A: monoclonal antibody LK2H10 diluted 1∶500 (Lab Vision/NeoMarkers/AHdiagnostics, Sweden, #MS-324-P). For detection of NESP55 and chromogranin A slides were microwave treated in TE buffer pH 9 (Dako Sweden, #S2367) for 10′ at 750 W, followed by 15′ at 350 W, then rinsed and run in a DAKO Autostainer plus which included a blocking step, 30 minutes of primary antibody incubation, 30 minutes amplification with the Dako REAL EnVision System (Dako Sweden, #K5007) and 10 minutes development. For negative controls the primary antibody was omitted. HIF1α and HIF2α: Primary antibodies were: mouse monoclonal antihuman HIF1*α* H1*α*67 (Neomarkers, Fremont, CA, USA), and 1/1000 rabbit polyclonal anti-mouse HIF2*á* PM8 antiserum, applied as described [33].

### Histological measurements

Distances from vascular stroma to regions of NESP55 immunreactivity were measured using digital images of NESP55-stained tumor sections and an index scale. Representative xenografts and clinical tumors were chosen based on presence of well demarcated regions of NESP55 immunoreactivity. Measure points were capillary endothelial layer and first layer of NESP55 immunoreactive tumor cells. A mean score, based on ten measurements, was made from each tumor.

### Statistical methods

Independent sample *t*-test was applied for determination of the significance of rt-qPCR data using SPSS software.

## Results

### Human fetal expression of NESP55

**Figure 1:**
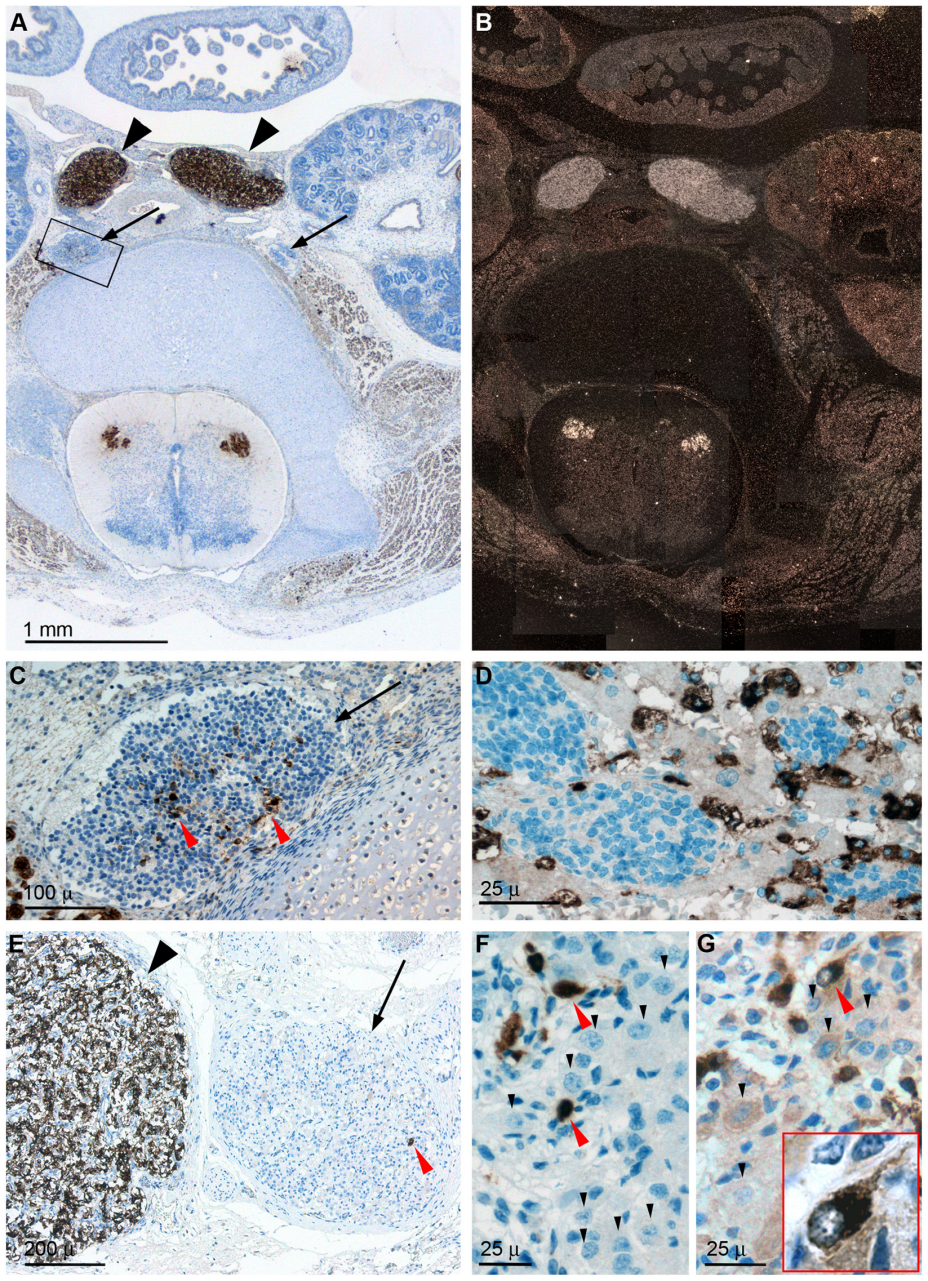
Sympathetic nervous system expression of NESP55 during human development. **A** and **B**: NESP55 immunohistochemistry (A) and *NESP55 in situ* hybridization (B) results from consecutive mid-abdominal sections of a nine week old fetus (developmental age) containing the organ of Zuckerkandl, i. e. the largest sympathetic paraganglia of the body, and sympathetic trunk ganglia. Other organs are: small intestine, kidneys, aorta, spinal cord, vertebra and paravertebral muscles. **C** and **D**: NESP55 immunoreactivity of sympathetic cell types in (C): an abdominal sympathetic trunk ganglion at age nine weeks, and (D): of the adrenal medulla at 18 weeks of development. Panel C is a high power view of boxed region in A and shows pericentric presence of NESP55 immunoreactive *small intensely fluorescent (SIF) cells*. Panel D shows adrenal tissue containing nests of sympathetic neuroblastic cells and NESP55 immunoreactive medullary chromaffin cells mixed with cortical cells. **E**–**G**: NESP55 immunoreactivity of para-adrenal sympathetic tissue of a term fetus. Panel E depicts a sympathetic paraganglion and an adjacent sympathetic ganglion. Panel F is a high power view of a sympathetic ganglion of the same specimen. G: Same ganglion as in F, tested with a four-fold higher anti-NESP55 antibody concentration. Insert is a high power view of an indicated SIF cell. *Symbols*: Large black arrowheads: sympathetic paraganglia; arrows: sympathetic ganglia, red arrowheads: SIF cells, small black arrowheads: ganglion cells.

In specimens representing developmental weeks six through eighteen NESP55 immunoreactivity was specific and strong for chromaffin constituents of the sympathetic nervous system – i.e. paraganglia cells (Fig. 1: A) chromaffin cells of the adrenal medulla (Fig. 1: D) and *small intensely fluorescent* (SIF) cells [34] of sympathetic ganglia (Fig. 1: C). During this late embryonic/early fetal phase of develoment, neuronal cells of sympathetic ganglia were morphologically primitive and lacked NESP55 immunoreactivity (Fig. 1: A and C), as was also the case throughout development for nests of primitive neuroblastic cells of the adrenal medulla (Fig. 1: D). At term, when sympathetic ganglion cells are morphologically more mature, NESP55 immunoreactivity was still essentially chromaffin-specific under standard testing (Fig. 1: E and F), although weak immunoreactivity was found in occasional ganglion cells. When testing at a four-fold increased anti-NESP55 antibody concentration a weak and general cytoplasmic immunoreactivity was evident at this stage of ganglion cell differentiation (Fig. 1: G). The small size of SIF cells, relative to ganglion cells, was also evident at this developmental stage (Fig. 1: F and G). Apart from their strong NESP55 immunoreactivity of both cell soma and cell process(es), SIF cells displayed a characteristic pattern of nuclear staining with punctate basophilia in a pale background (Fig. 1: G), characteristic of chromaffin cells [35]. NESP55 expressing cell types outside the sympathetic nervous system were: motor neurons of the spinal medulla, striated muscle, metanephric blastema, and macula densa-like cells associated with the glomerular tuft (Fig. 1: A and data not shown). NESP55 *in situ* hybridization revealed an identical cell type-specific pattern of expression to the immunohistochemical analyses (Fig. 1: B). Immunoreactivity was almost completely abolished following epitope-adsorption, with no evident difference in cellular specificity of the remaining weak signal (data not shown). No immunoreactivity was seen with non-blocked antibodies applied to brain sections from *nesp55* knock-out mice (data not shown). Therefore the NESP55 antibodies are both specific and sensitive for detection of NESP55.

**Figure 2:**
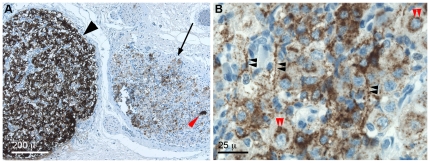
Chromogranin A immunoreactivity of the human fetal sympathetic nervous system. **A**: Sympathetic paraganglion and adjacent sympathetic ganglion of a term fetus. Same region as shown in Figure 1 panel E, analyzed in a consecutive section. **B**: High-power view of a sympathetic ganglion from the same section. *Symbols*: black arrowhead: sympathetic paraganglion; arrow: sympathetic ganglion, red arrowhead: SIF cell. Black double arrowheads indicate axonal stainings and red double arrowheads indicate perinuclear stainings of ganglion cells.

These findings were compared with chromogranin A immunoreactivity in consecutive sections. Analogous to NESP55, immunoreactivity to chromogranin A by chromaffin cell types was intense (Fig. 2: A). In contrast, significant chromogranin A immunoreactivity of sympathetic neuronal cell processes was found at all stages of development, establishing that chromogranin A is a less specific marker for chromaffin cell types than NESP55 (compare Fig. 2 and Fig. 1: E and F). Furthermore, the cytoplasm of morphologically mature ganglion cells displayed a different staining pattern for chromogranin A, with patchy and mainly peri-nuclear signal (compare Fig. 2: B and Fig. 1: G).

These results indicate that NESP55 is a sensitive chromaffin marker in the early human sympathetic nervous system, with a specificity superior to that of chromogranin A.

### Expression of NESP55 and other genes of interest in neuroblastoma under anaerobic cell culture

In order to test whether *NESP55* expression was influenced by hypoxia in neuroblastoma, Kelly, SK-N-BE(2), SH-SY5Y, SK-N-FI, and LAN-5 cells were tested with quantitative real-time PCR after exposure to anaerobic conditions. Up-regulation of *NESP55* mRNA levels was seen in all cell lines, ranging from 3- to 53-fold (Table 1).

**Table 1:** Effects of anoxia on expression of *NESP55* and of other genes in five neuroblastoma cell lines.

	Kelly	SK-N-BE(2)	SH-SY5Y	SK-N-FI	LAN5
***NESP55***	**4.5** (.34**)	**21** (3.3*)	**53** (.97***)	**14** (1.4*)	**3.0** (.36*)
***IGF2***	**2.1** (.66 ns)	**9.3** (.83***)	**577** (40***)	**11** (.78***)	**1055** (177**)
***HIF2α***	**0.39**(.011***)	**1.6** (.16**)	**1.3** (.052***)	**3.0** (.29**)	**0.61** (.17 ns)
***HIF1α***	**0.25** (.014***)	**0.22**(.015***)	**0.53** (.085**)	**0.20** (.008***)	**0.25**(.023***)
***NOTCH1***	**13** (1.3***)	**0.50**(.039***)	**9.5** (2.9*)	**18** (2.0***)	**4.4** (.63**)
***HES1***	**0.11** (.015***)	**0.08**(.006***)	**0.17**(.013***)	**0.17** (.013***)	**0.08**(.005***)
***ID2***	**0.09** (.008***)	**0.16**(.011***)	**0.47** (.15***)	**0.39** (.021***)	**0.40**(.034***)
***CgA***	**0.13** (.089***)	**0.40**(.083***)	**0.87** (.18 ns)	**0.30** (.024***)	**0.63**(.039***)

Results are relative to levels of control cells cultured in parallel at ambient air oxygen tension (“normoxia”) and represent means of two quantitative real-time PCR analyses, each performed in triplicate. *Symbols*: *: p<.05; **: p<.01; ***: p<.001; ns: non-significant difference. *CgA*: chromogranin A. Numbers within brackets refer to standard error of mean.

We also examined expression of other genes of interest (Table 1): *IGF2* was massively (>500-fold) up-regulated by hypoxia in SH-SY5Y and LAN-5 cells from very low levels under standard conditions. Despite higher levels of basal expression in the remaining three cell lines, two of these also displayed robust hypoxic *IGF2* up-regulation, comparable to that of *NESP55*. HIF regulation is primarily through oxygen-dependent hydroxylation of the alpha subunits, which enables their efficient degradation in the presence of oxygen [36]. HIF-1α and HIF-2α are non-redundant, have differential effects on target genes and are expressed in different cell types [33], [37], [38]. Expression of *HIFα* genes is usually not greatly influenced by hypoxia at the mRNA level [39]. Interestingly, in neuroblastoma cells we did find a clear oxygen dependence of these transcripts: *HIF2α* transcript levels were significantly induced by anoxia in SK-N-BE(2), SH-SY5Y, and SK-N-FI cells, whereas *HIF1α* was down-regulated in all five neuroblastoma cell lines. A similar finding of interest was hypoxic down-regulation of *β-actin* expression in all cell lines, which excluded its use as an internal standard for normalization of our values (data not shown). Furthermore, our data indicated that both *Hairy Enhancer of Split homolog-1* (*HES1*), and *Inhibitor of DNA binding 2 (ID2)* transcripts were down-regulated by anaerobic hypoxia in all cell lines, whereas *NOTCH1* was up-regulated in four of the cell lines (Table 1). However, the real-time PCR data indicated very low expression levels of *NOTCH1*. These three genes were tested because of their previously reported roles in modulating neuroblastoma phenotype in association with reduced cellular oxygen tension [40]. The expression of *CHROMOGRANIN A* was down-regulated by hypoxia in four of the cell lines under these culture conditions (Table 1).

### Expression of NESP55 in tumor xenografts, relationship to hypoxia markers and to vascular stroma

Next, we tested whether regional hypoxia in solid tumours would induce expression of NESP55. Therefore, we generated xenograft tumors in immunodeficient mice from the cell lines tested with real-time PCR. Serial sections were prepared and the spatial distribution of NESP55 immunoreactive cells was studied in relation to the vascular stroma and to expression of markers for hypoxia and phenotype: nuclear HIF2α immunoreactivity was selected as an operational definition of a cellular response to hypoxia and expression of *VEGFA* and of *IGF2* were used as independent hypoxia markers, previously validated in neuroblastoma [11], [12], [41]. The mRNA expressions of *NESP55*, *HIF1α*, and *HIF2α* were also studied with in situ hybridization. In addition, chromogranin A expression was studied both immunohistochemically and with in situ hybridization. Finally, we studied the distribution of *Growth-Associated Protein 43 (GAP 43)* expressing cells, which is a marker for axonal growth [42] and an established marker for an early sympathetic neuronal phenotype in neuroblastoma [12], [43]. These histological findings are summarized in Table 2.

**Table 2:** Histological evidence of hypoxia-dependence of expression of NESP55 and of other genes in neuroblastoma xenografts.

	Kelly	SK-N-BE(2)	SH-SY5Y	SK-N-FI	LAN5
***Candidate markers of phenotype***					
NESP55-IHC	**⇑**(sp)	**⇑**(sp)	**⇑**(sp)	⇑(gr/w)	⇑*
*NESP55-ISH*	ND	ND	**⇑**(gr/str)	ND	ND
*IGF2-ISH*	**⇑**(sp)	**⇑**(gr/str)	**⇑**(sp)	**⇑**(gr/str)	**⇑**(sp)
*HIF2∼α-ISH*	⇑(gr/w)	**⇑**(gr/str)	**⇑**(gr/str)	**⇑**(sp)	⇑(gr/w)
*GAP 43-ISH*	⇓(partial)	⇓(partial)	**⇓**(complete)	⇓(partial)	** = **
CgA-IHC	** = **	** = **	** = **	**⇑**(sp)	** = **
*CgA-ISH*	** = **	** = **	** = **	**⇑**(gr/str)	** = **
*HIF1∼α-ISH*	ND	ND	⇓(partial)	** = **	ND
*β-actin-ISH*	ND	=	⇓(partial)	ND	ND
***Hypoxia markers***					
HIF2αIHC	⇑(ref)	⇑(ref)	⇑(ref)	⇑(ref)	⇑(ref)
HIF1αIHC	⇑(sp)	⇑(sp)	⇑(sp)	⇑(sp)	⇑(sp)
*VEGFA-ISH*	⇑(sp)	⇑(sp)	⇑(sp)	⇑(sp)	⇑(sp)

Nuclear HIF2α immunoreactivity was used as operational definition of hypoxia. Serial sections were used to compare the different results.

*Abbreviations and symbols*: IHC: immunohistochemistry; ISH: *in situ* hybridization; CgA: chromogranin A.

**⇑**(sp): positivity specific for regions of chronic hypoxia.

**⇑**(gr/w): weak positive gradient of expression in regions of chronic hypoxia but expression not specific to these regions.

⇑*: few positive cells, but exclusively in regions of chronic hypoxia.

ND: not analyzed.

**⇑**(gr/str): strong positive gradient of expression in regions of chronic hypoxia but expression not specific to these regions.

**⇓**(partial): partial down-regulation in regions of chronic hypoxia.

**⇓**(complete): complete down-regulation in regions of chronic hypoxia.

**= **: no discernible chronic hypoxia-dependence of expression intensity.

**⇑**(ref): reference method for identification of regions of chronic hypoxia.

**Figure 3:**
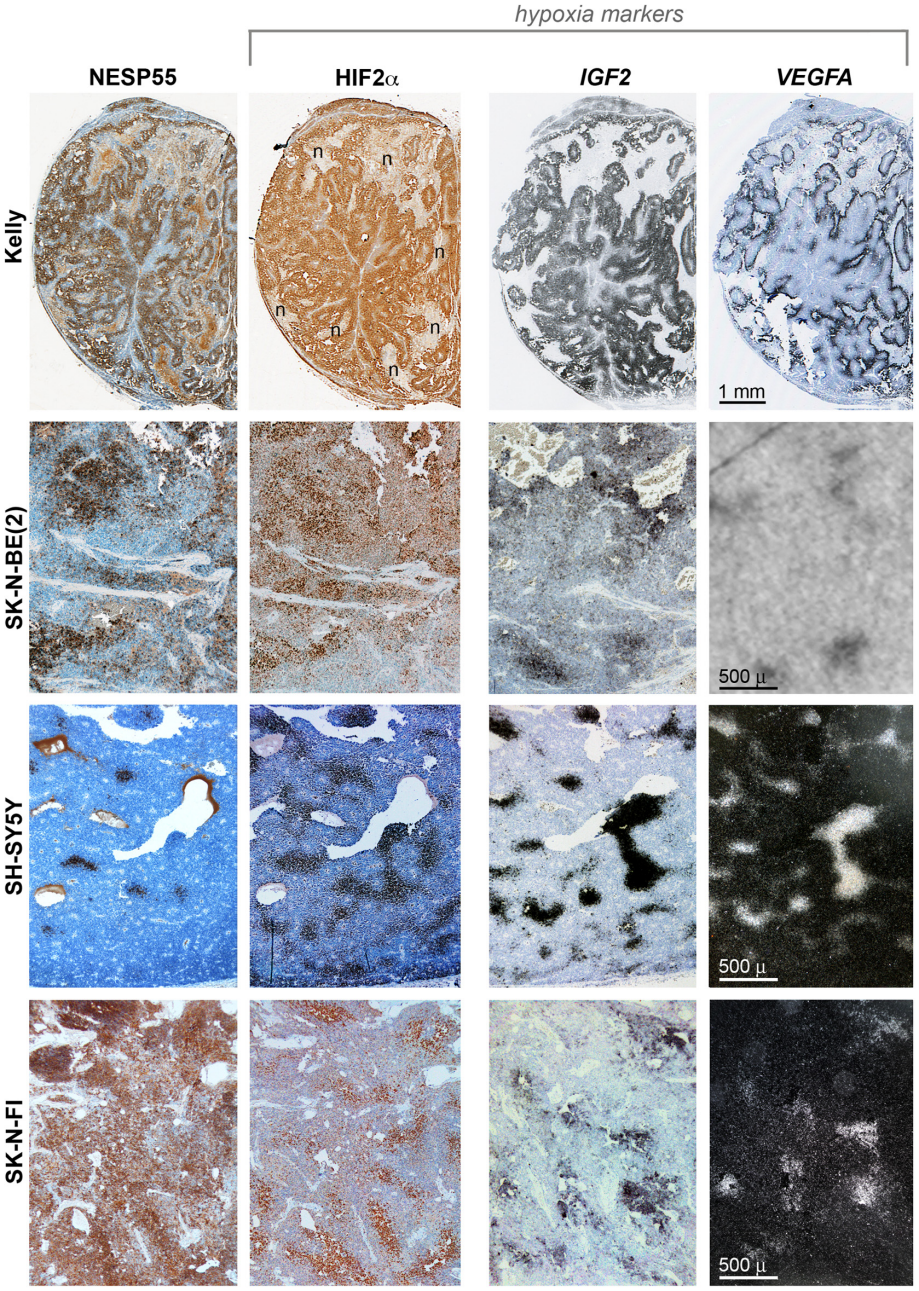
NESP55 immunoreactivity and histological evidence of hypoxia in neuroblastoma xenografts. Columns represent NESP55/HIF2α immunoreactivities and *IGF2*/*VEGFA in situ* hybridization (ISH) results, as shown. Rows represent different cell lines, as described. In each row the same tumor region is shown for the different analyses. NESP55/HIF2α results are produced from consecutive sections. *IGF2*/*VEGFA* results are from consecutive sections that are adjacent, but not consecutive, to the former sections. HIF2α immunoreactivity and *IGF2*/*VEGFA* expressions were chosen as markers for cellular hypoxia. *IGF2* ISH results and *VEGFA* ISH results for Kelly are shown in brightfield view (silver grains appear in black). *VEGFA* expression in SK-N-BE(2) is represented by an x-ray film autoradiography and *VEGFA* ISH results for SH-SY5Y and SK-N-FI xenografts are shown in darkfield view (silver grains appear in white). *Symbol*: n: areas of necrosis.

**Figure 4:**
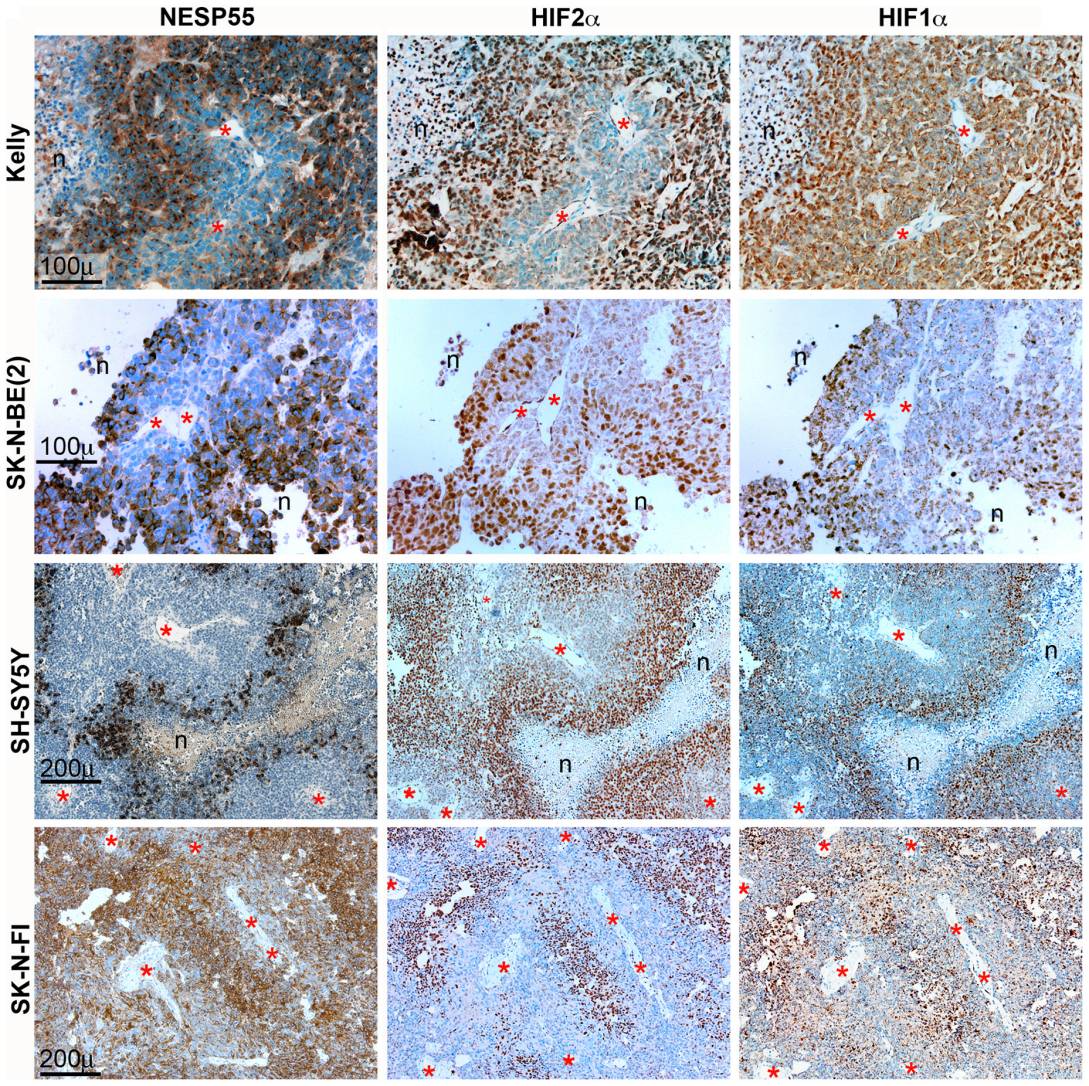
Arrangements of NESP55, HIF2α and HIF1α immunoreactive cells in neuroblastoma xenografts related to fibro-vascular stroma. Columns represent the different immunohistochemical analyses, as indicated, and rows show a tumor region, analyzed in consecutive sections, of mouse xenografts derived from four different neuroblastoma cell lines, as indicated. *Symbols*: n: areas of necrosis; asterisks: vascular lumina.

As exemplified in Figure 3, immunoreactivity for NESP55 was clearly heterogeneous in the various tumor xenografts and the proportion of cells that were labelled differed between tumors. NESP55 immunoreactive regions coincided strikingly with regions displaying nuclear immunoreactivity to HIF2α (Fig. 3), implying that they were hypoxic. The concordance between *IGF2* and *VEGFA* expressing regions and their striking overlap with regions of nuclear HIF2α accumulation (Fig. 3) is consistent with each of these being activated by hypoxia. SK-N-FI was the only cell line generating tumors with more generalized NESP55 immunoreactivity. In this tumor type more intense staining was observed in regions with HIF2α staining (Fig. 3). Examination under higher magnification showed that the increased NESP55 immunoreactivity in hypoxic regions of SK-N-FI-derived tumors was contributed to by increased signal intensity in each cell, and an increased proportion of positive cells (Fig. 4). In other tumors there was also strong spatial correlation between NESP55 immunoreactivity and nuclear HIF2α at the cellular level (Fig. 4 and Table 2). It was also evident that NESP55 and HIF2α immunoreactivities were dependent upon tumor cell distance from blood vessels (Fig. 4). Mean distances to NESP55 immunoreactive cells, as previously defined, were 50 µm, 93 µm and 50 µm for xenografts derived from Kelly, SH-SY5Y and SK-N-BE(2) cells, respectively. At further distances from the blood vessels the NESP55/HIF2α positive regions frequently bordered on areas of tumor necrosis (Fig. 3: Kelly and Fig. 4: Kelly, SK-N-BE(2) and SH-SY5Y). This distribution of HIF2α immunoreactive tumor cells, in combination with the well-characterised hypoxia-dependent stabilisation of HIF2αare consistent with the notion that regions with combined NESP55 and HIF2α immunoreactivity represent chronically hypoxic tumor cells, caused by the limited range of oxygen diffusion from the microvascular circulation.

Nuclear immunoreactivity of HIF1α coincided with that of HIF2α in all neuroblastoma xenografts (Fig. 4, Table 2), albeit with lower intensity which was particularly evident in tumors derived from Kelly cells. The strikingly similar distribution of immunoreactivity for HIF1α and HIF2α in neuroblastoma supports the validity of our hypoxia markers.

### NESP55-independent evidence for a hypoxia-dependent shift of sympathetic phenotype in neuroblastoma

**Figure 5:**
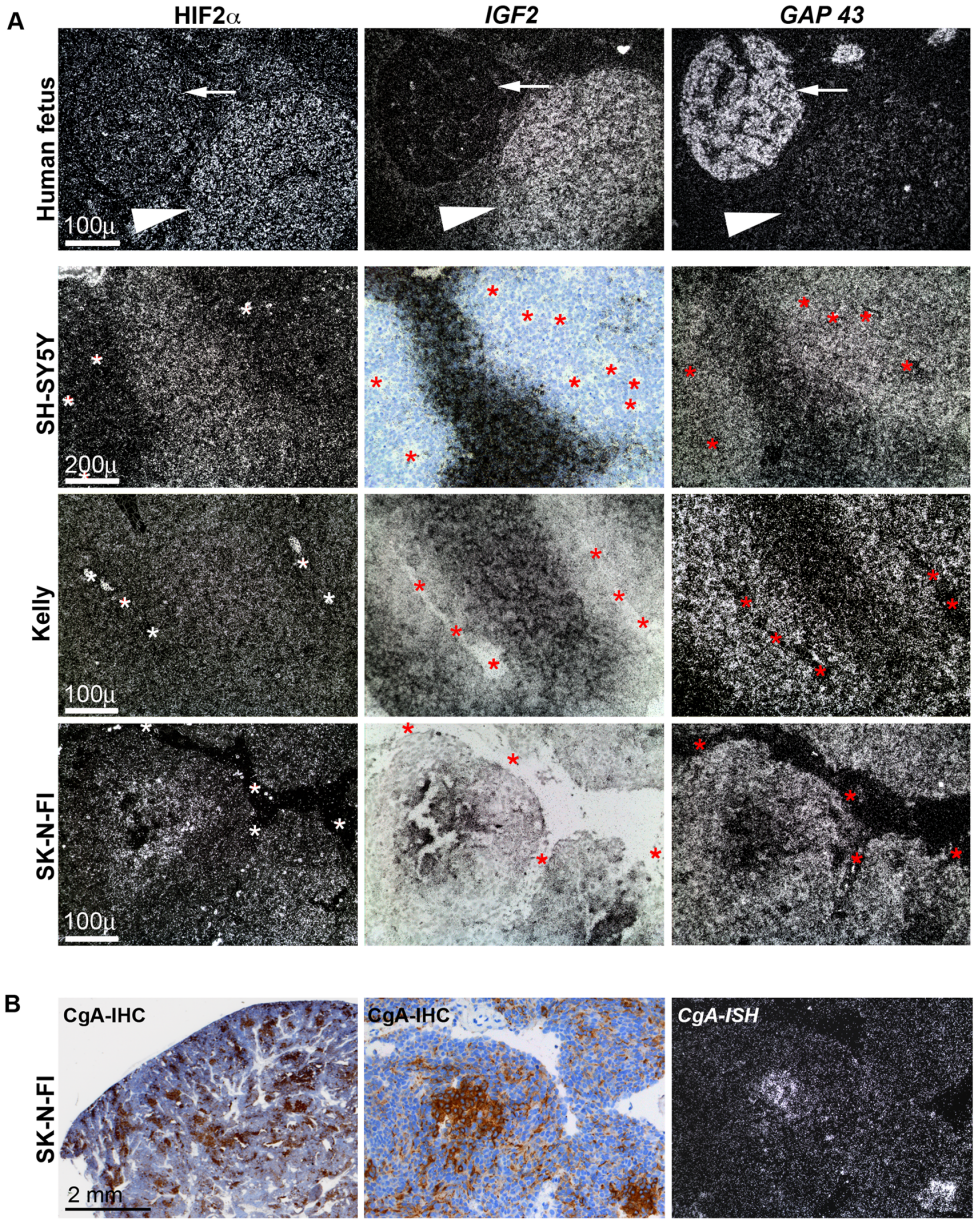
NESP55-independent evidence of a chromaffin hypoxic phenotype in neuroblastoma xenografts. **A**: Columns show *HIF2*α, *IGF2* and *GAP 43 in situ* hybridization results, as indicated. The specificities of these expressions within the early sympathetic nervous system are shown in the upper row, representing consecutive sections of a 12 week fetal specimen containing a sympathetic ganglion and a sympathetic paraganglion, as indicated. The rows below represent consecutive sections from three different neuroblastoma xenograft tumors, as indicated. Silver grains appear in black in panels showing *IGF2* expression (brightfield illumination). In all other panels silver grains appear in white (darkfield illumination). *IGF2* ISH is used here as a combined marker for a chromaffin phenotype and for tumor hypoxia. *Symbols*: arrows: sympathetic ganglion; arrowheads: sympathetic paraganglion; asterisks: vascular structures. **B**: Chromogranin A expression in a SK-N-FI neuroblastoma xenograft. Left and middle panels depict chromogranin A immunoreactivity at two different magnifications. Right panel shows a *chromogranin A in situ* hybridization result in darkfield view from a consecutive section. Middle and right panels represent the same tumor region as depicted in the bottom row of Fig. 5: A.

Interestingly, areas of predicted hypoxia were associated with increased mRNA expression of *HIF-2α* in tumor xenografts and there was also evidence of this in several lines in our anaerobic culture studies (Fig. 5: A; Tab. 1 and 2). Together with *IGF2* expression, this observation provides some independent support for a neuroendocrine tumor phenotype of hypoxic neuroblastoma cells in view of the chromaffin-specific expression of *HIF2α* [44], [45] and *IGF2* [8], [9] in fetal tissues (also shown in Fig. 5: A). A complementary sign of a shift in phenotype in hypoxic regions was provided by *GAP 43 in situ* hybridization. This marker of an early neuronal phenotype was markedly decreased in hypoxic tumor regions, parallel to induced *HIF2α* and *IGF2* (Fig. 5: A and Table 2). A more traditional neuroendocrine feature in regions of tumor hypoxia was observed in tumors generated from SK-N-FI cells: in this tumor type chromogranin A expression was hypoxia-dependent, both at the protein and the transcriptional levels (Fig. 5: B and Table 2).

### Expression of NESP55 in clinical neuroblastoma and ganglioneuroma

**Figure 6:**
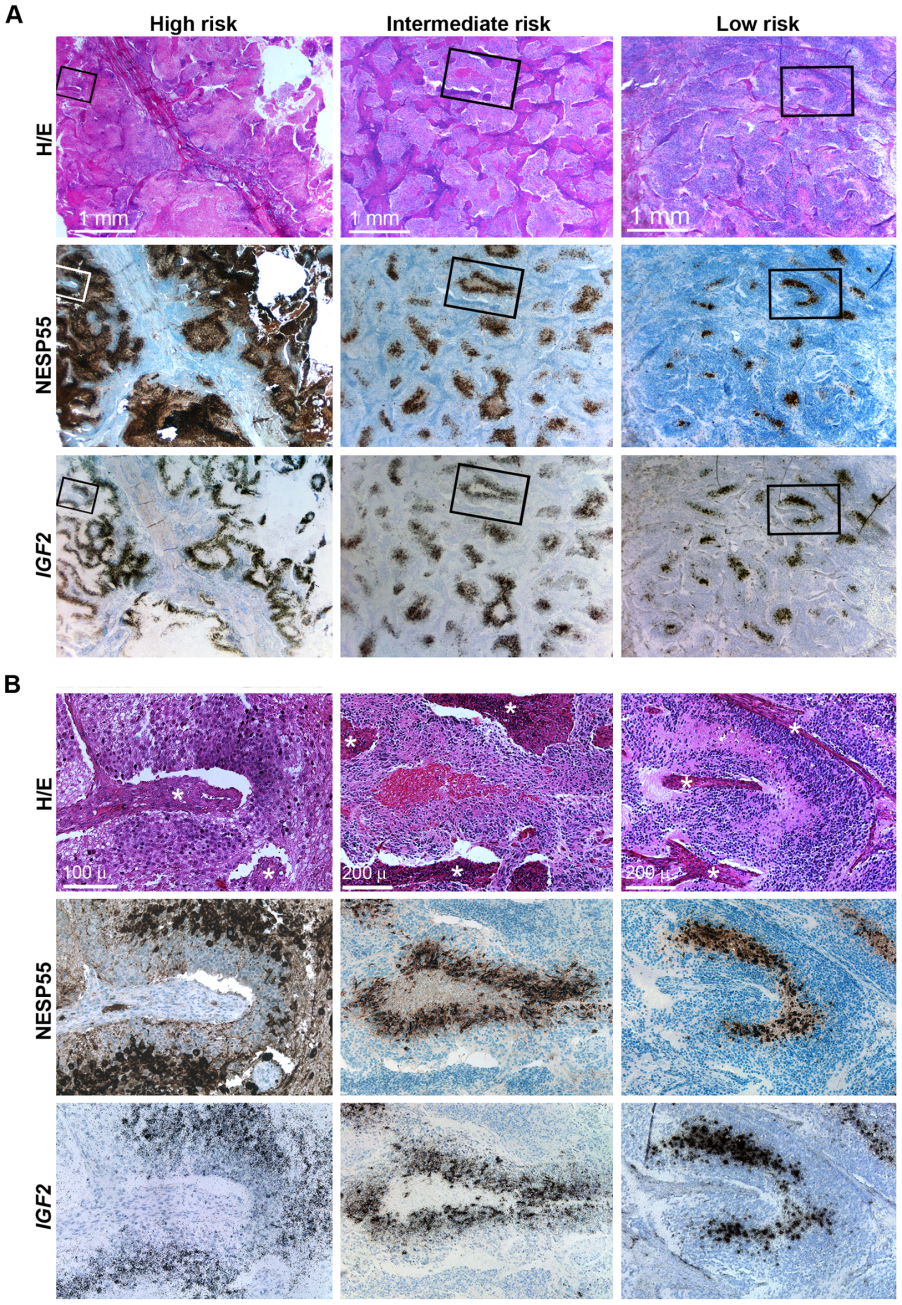
NESP55 immunoreactivity and *IGF2* expression in clinical variants of neuroblastoma. **A**: Low power magnification: First column: abdominal stage 4 tumor with a poor patient outcome. Second column: abdominal stage 3 tumor of an adolescent. Patient outcome was poor due to complications after myeloablative therapy. Third column: abdominal infant stage 3 tumor with a favourable clinical outcome. All specimens were obtained prior to start of chemotherapy. **B**: High power views of the same sections (boxed regions in A): *Symbol*: asterisks: fibro-vascular stroma.

**Figure 7:**
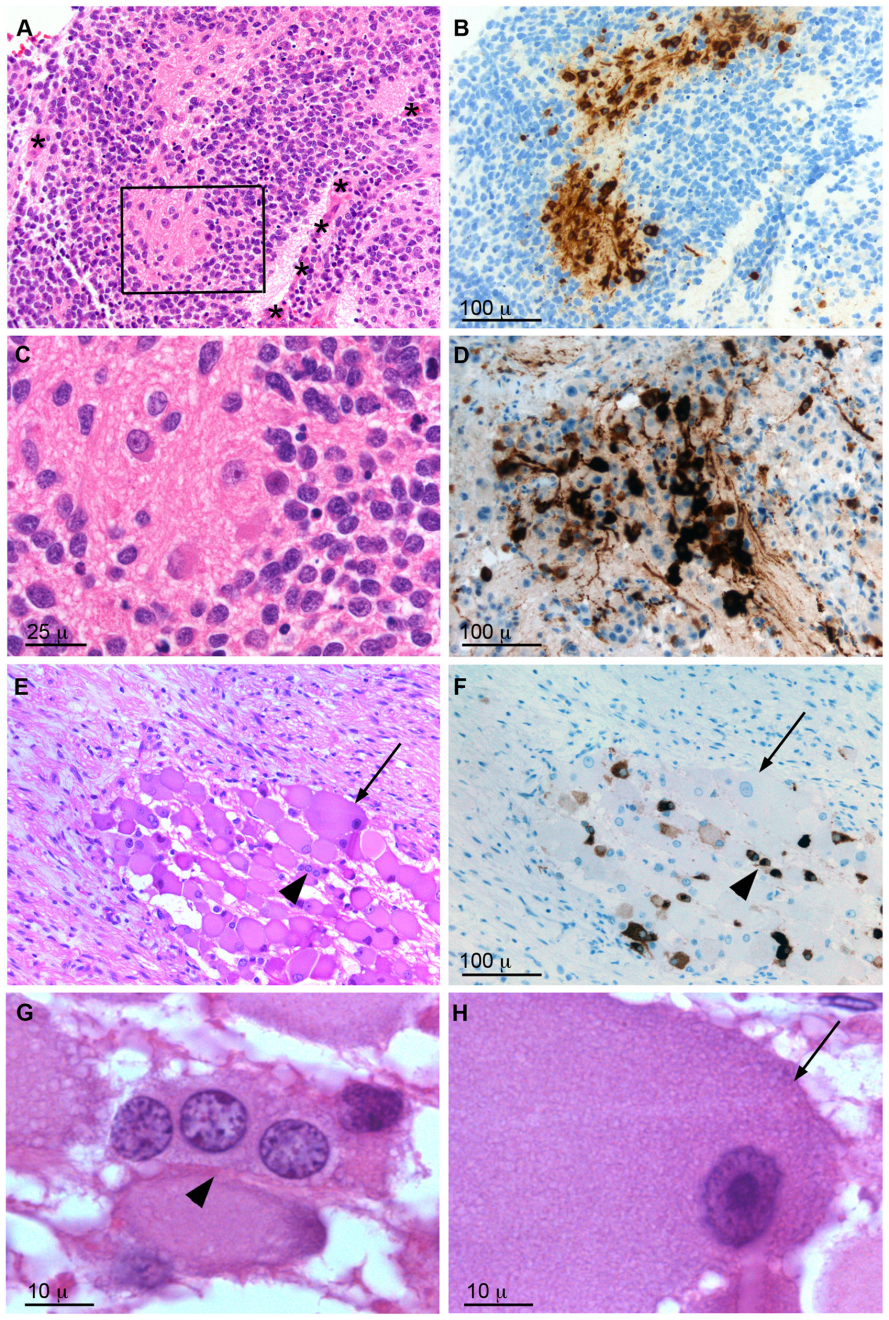
Morphological features of NESP55 immunoreactive cells in clinical neuroblastoma and in ganglioneuroma. **A–C**: Favourable outcome, stroma-poor infant stage 3 neuroblastoma. Boxed region in panel A is shown in panel C. **D**: prognostically unfavourable, stroma-poor adolescent stage 4 neuroblastoma. **E–H**: Stroma-rich ganglioneuroma. G and H show the respective nuclear morphologies of cells indicated in E. Panels A, C, E, G and H are haematoxylin-eosin stanings and panels B, D and F show NESP55 immunoreactivity. Panels B and F are from sections consecutive to A and E, respectively. *Symbols*: asterisks: fibro-vascular stroma, arrowheads: indicate a group of three NESP55 immunoreactive cells, arrows: indicate a large tumor cell lacking NESP55 immunoreactivity.

We then proceeded to examine NESP55 expression and its relation to hypoxia in clinical specimens of neuroblastoma. Expression of *IGF2* and *VEGFA* were used as markers of a hypoxic response in our histological testing of these specimens. All but two tumors displayed NESP55 immunoreactive cells (Table 3). *NESP55* in situ hybridization showed results identical to those of NESP55 immunohistochemistry, supporting the accuracy of the immunoanalysis (data not shown). The proportions of immunoreactive tumor cells varied between tumors and expression was regional with an abrupt onset at distances varying from 87 to 160 µm (median 115 µm) from the vascular stroma, which was strikingly coordinated with expression of *IGF2* (Fig. 6: A and B). Based on co-expression with *VEGFA* and/or *IGF2*, cells expressing NESP55 were identified as hypoxic in seventeen of the 24 specimens in which there was positive signal (Table 3). We did not observe a correlation between hypoxic NESP55 expression and the prognostic category of neuroblastoma. Interestingly we observed hypoxic NESP55 expression in tumors from all categories suggesting that there is not a strong relationship to prognostic category, but the sample size was relatively small and so we cannot exclude such an association (Table 3; exemplified in Fig. 6). Specific locations and cytological characteristics of NESP55 positive tumor cells were evident: typically, and in analogy to the xenografts, NESP55 positive cells were positioned at a distance from the fibrovascular stroma (Fig. 6: B and Fig. 7: B). Morphological characteristics of these cells were: an enlarged and eosinophilic cell soma (Fig. 7: A and C), nuclear enlargement and pallor (Fig. 7: C), differences in cell orientation (Fig.7: A and B), and more abundant processes and neuropil, as compared to adjacent tumor cells (Fig. 7: A–D). The nuclear characteristics were reminiscent of chromaffin cell types (compare Fig. 1: G) and differed clearly from those of mature ganglion cells. In ganglioneuroma small NESP55 positive cells with the same nuclear characteristics (Fig. 7: E–G) were found mingled with larger tumor cells with weaker, or absent, NESP55 immunoreactivity and a ganglion cell-like nuclear morphology (Fig. 7: E, F and H). Hence, we found evidence for the co-existence of two types of differentiation in stroma-rich tumors.

**Table 3:** Hypoxia-dependence of NESP55 immunoreactivity in clinical variants of neuroblastoma and in ganglioneuroma.

CLINICAL DATA			NESP55	expressed	NESP55	not expressed§
			*Hypoxia-dependent NESP55 expression* (NESP55/*IGF2*/*VEGFA* co-expression)	*No evidence for hypoxia-dependent NESP55 expression* (*IGF2*/*VEGFA* not expressed)§	*Signs of hypoxia* (*IGF2*/*VEGFA* expressed	*No signs of hypoxia* (*IGF2*/*VEGFA* not expressed)§
Clinical group	n	Alive				
Ganglioneuroma	2	2	1(0*)	1(0*)	-	-
Infant stage 1**–**3	4	4	3(0*)	-	-	1(0*)
Stage 4S	1	1	-	-	1(0*)	-
Stage 3**–**4>18 mo	18	1	12(3*)	6(6*)	-	-
Stage 1>18 mo	1	0	1(0*)	-	-	-
**TOTAL**	**26**	**8**	**17**	**7**	**1**	**1**

NESP55 expression was evaluated by immunohistochemistry and hypoxia dependence by its co-expression with *VEGFA* and/or *IGF2*, as determined with *in situ* hybridization performed on sections consecutive to those used for the NESP55 analyses.

*Abbreviations and symbols*: **n**: number of cases;

§ negative results are based on two independent experiments;

*number of tumors subjected to chemotherapy prior to sampling; >18 mo: more than 18 months of age at diagnosis.

**Figure 8:**
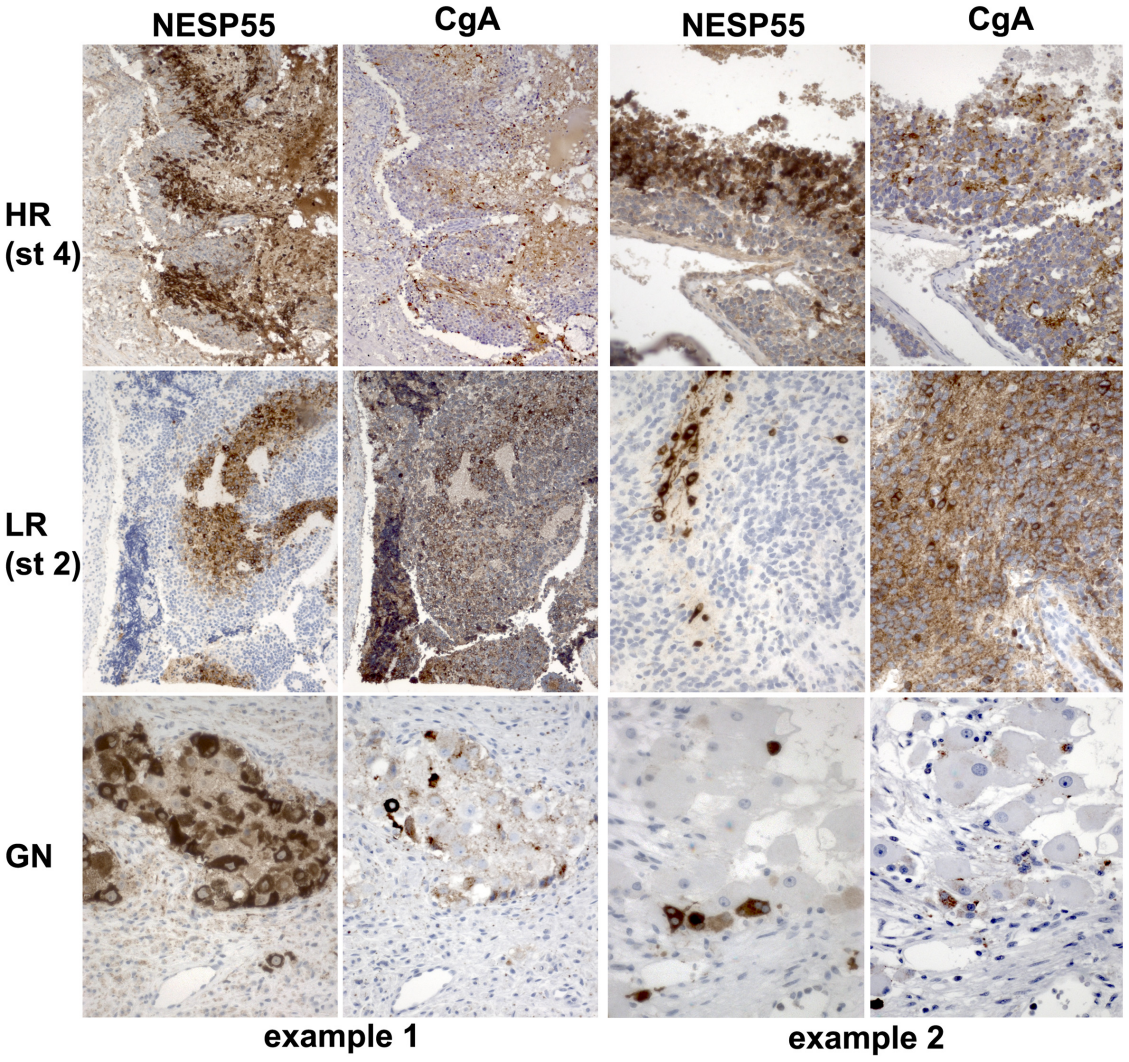
Regional co-expression of NESP55 and chromogranin A in clinical subtypes of neuroblastoma. Each row represents a clinical subset of sympathetic neuroblastic tumors showing NESP55 and chromogranin A immunoreactivity in consecutive sections, as exemplified in two separate tumors. The upper two rows represent tumors with a stroma-poor histology and bottom row shows nests of tumor cells within a stroma-rich environment from two ganglioneuromas. Note a more general membraneous/neuritic chromogranin A immunoreactivity in some of the specimens, as compared to cytoplasmic staining for chromogranin A in regions of NESP55 immunoreactivity. *Abbreviations*: CgA: chromogranin A; HR: high-risk neuroblastoma according to INRG criteria, LR: low-risk neuroblastoma according to INRG criteria; st 4: INSS stage 4 (metastatic); st 2: INSS stage 2; GN: ganglioneuroma

Although NESP55 immunoreactivity and *IGF2* expression were tightly linked in most tumors, this correlation was not observed in seven of the NESP55 positive tumors. Tumors from patients who had already received treatment were strikingly over-represented in this category (Table 3), so it is possible that treatment-related effects had altered gene expression patterns, resulting in decreased *VEGFA* and *IGF2 in situ* hybridization results in these tumors. However, two of them were particularly interesting because of general NESP55 positivity, independent of distance from fibro-vascular stroma. This would support the possibility that hypoxia-independent NESP55 expression does occur in some neuroblastomas. Clinically, these two tumors were a metastatic neuroblastoma and a ganglioneuroma. In general, chromogranin A immunoreactivity was also observed in hypoxic tumor cells but the gradients in expression were less marked than NESP55 (Fig. 8).

### NESP55 expression in pediatric brain tumor specimens and cell lines

We considered the possibility that NESP55 expression may be induced in other tumors of neuronal origin in response to hypoxia, which might suggest it was a general response to hypoxia as opposed to a context-specific phenomenon consistent with chromaffin differentiation. 35 pediatric brain tumors were therefore tested for presence of chronically hypoxic regions by identification of graded *VEGFA* signals with ISH and, when present, NESP55 IHC was performed on consecutive sections. None of the twelve tumors (7 gliomas, 3 supratentorial PNETs, 2 ependymomas) tested with IHC displayed any NESP55 immunoreactivity in regions with hypoxia-induced *VEGFA* expression, or elsewhere in the tumors. The other 23 tumors did not display gradients of *VEGFA* expression and were therefore not tested with NESP55 IHC. We also tested *NESP55* expression under anaerobic conditions in the human medulloblastoma cell line D324 Med and in the human supratentorial PNET cell line PFSK1 indicating insignificant (1.2-fold; standard error of mean .035) or minor (2.4-fold; standard error of mean .178; p<.001) induction, respectively.

## Discussion

### Fetal chromaffin physiology and NESP55 as a chromaffin marker

The mammalian sympathetic nervous system displays a fetal-specific organization of the chromaffin compartment with the major constituents being bulky retroperitoneal paraganglia and abundant SIF cells within sympathetic ganglia [34], [35]. These extra-adrenal chromaffin cells play important roles in fetal oxygen homeostasis [46] and undergo involution postnatally [35]. We show here that NESP55 is a marker for a chromaffin phenotype in the developing human sympathetic nervous system. In the earliest stages studied, when ganglion cells are still morphologically immature, its expression was strictly limited to paraganglia cells, SIF cells, and chromaffin cells of the adrenal medulla. Immunohistochemical and *in situ* hybridization data both indicated abundant expression at all developmental stages studied, starting from the late embryonal phase when paraganglia begin to form (developmental age six weeks). This pattern of expression within the developing human sympathetic nervous system strongly resembles that of *IGF2* [8], [9] and defined NESP55 as a clearly superior marker for a sympathetic neuroendocrine phenotype when compared to chromogranin A.

### Evidence for a chromaffin hypoxic tumor phenotype in neuroblastoma provided by NESP55 and by independent markers

In neuroblastoma NESP55 was commonly expressed and found mainly in association with diffusion-limited tumor hypoxia. This provides strong support for our previous suggestion that neuroblastoma cells respond to hypoxia by shifting towards a more mature neuroendocrine phenotype. In the current study we have taken an exhaustive approach to address this phenomenon, exemplified by the following: First, the specificity and sensitivity of the anti-NESP55 antibodies we developed were confirmed via testing epitope adsorbed antibodies, precise correspondence with *NESP55 in situ* hybridization, and lack of NESP55 immunoreactivity in brain tissues from *nesp55* knock-out mice. Second, neuroblastoma cell line experiments showed that anoxia strongly increases expression of *NESP55* mRNA in all five cell lines tested, and that presence of NESP55 protein in the corresponding xenografts correlated with distance from blood vessels. Third, independently of our NESP55 data, other changes in gene expression support a chromaffin phenotype of hypoxic neuroblastoma cells: up-regulation of *IGF2* and of *HIF2α*, in particular, was strong evidence of this, as chromaffin-specific expression within the sympathetic nervous system of both of these genes was shown both in this and in previous investigations [9], [44], [45]. Parallel to this, hypoxic down-regulation of *GAP 43* in the xenografts was evidence in support of loss of neuronal tumor characteristics. Although not studied here, this hypoxia-dependence of *GAP 43* expression applies also to clinical neuroblastoma/ganglioneuroma [12]. In the clinical specimens studied here, *IGF2* co-expression with NESP55 was seen in the majority of cases and chromogranin A/NESP55 co-expression was frequent and was also observed in xenografts of SK-N-FI cells. Fourth, independent markers for hypoxia were employed and were congruent with NESP55 expression at the cellular level in tumor tissues. Fifth, we observed that NESP55 expression was tightly linked to morphological signs of tumor maturation in clinical tumors, with features resembling those of SIF cells [34]. Sixth, we excluded the possibility that hypoxic NESP55 immunoreactivity is a general response to hypoxia by testing some other pediatric tumors of central nervous system origin and examining *NESP55* expression in two non-neuroblastoma brain tumor-derived cell lines. We have also tested 48 pediatric brain tumors of different types with *in situ* hybridization for *IGF2* expression. None of the tested specimens displayed a hypoxic pattern of *IGF2* expression (unpublished data). These experiments are consistent with hypoxic expression of NESP55 and of *IGF2* representing markers for chromaffin differentiation in neuroblastoma tissue.

### Implications for concepts of differentiation in neuroblastoma and for neuroblastoma pathology

**Figure 9:**
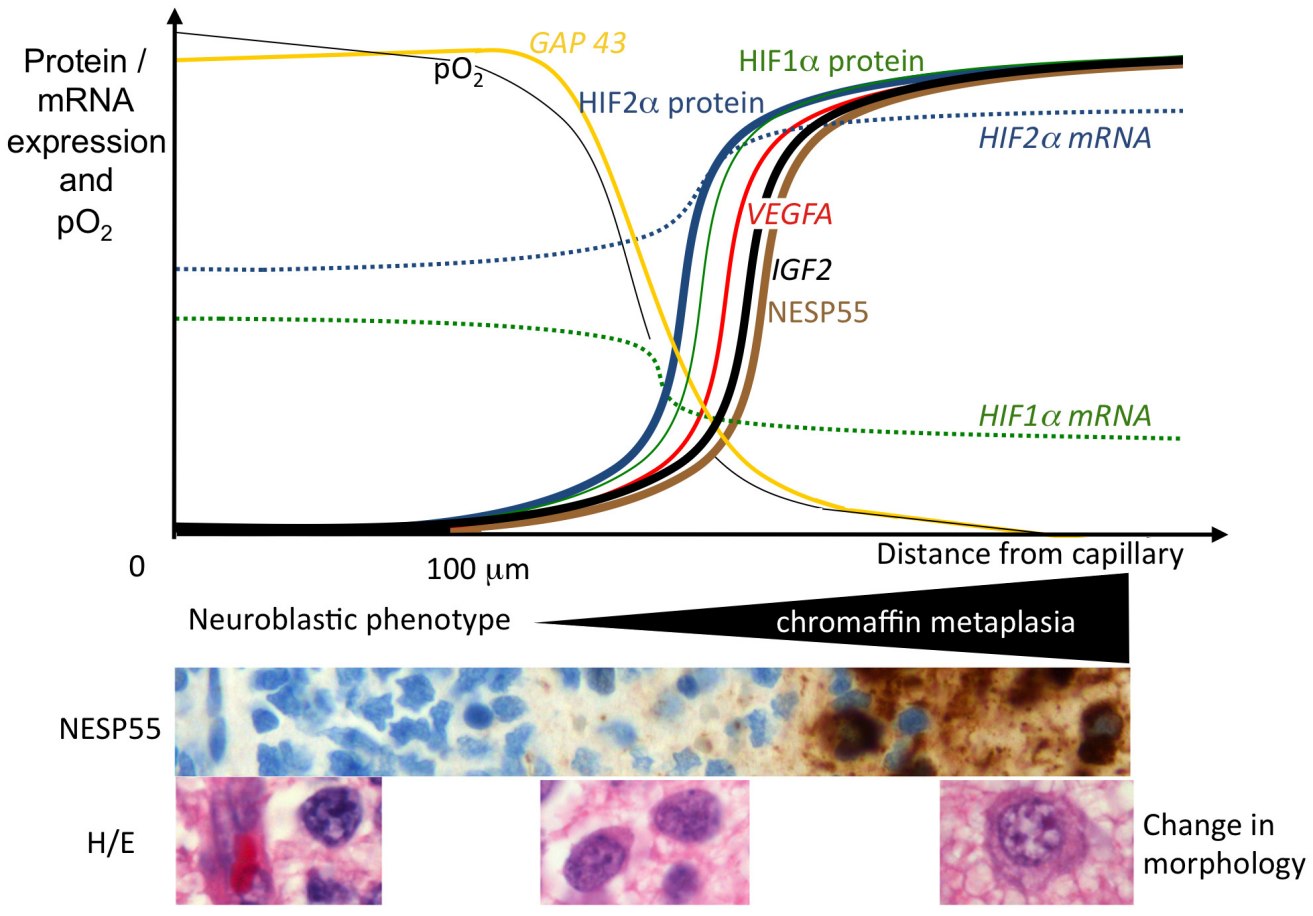
Schematic summary of the studied effects of diffusion-limited hypoxia in neuroblastoma. x-axis symbolizes the distance from tumor capillary and y-axis symbolizes the relative levels of marker gene expression and of oxygen tension (pO_2_). Photo images exemplify the microvascular-dependence of NESP55 immunoreactivity and of changes in cell morphology. Photo images are from the same tumor region of an infant neuroblastoma, taken from consecutive sections. Black triangle symbolizes the region of increasing chromaffin metaplasia parallel to decreasing tissue oxygen tension.

The present data support the conclusion that chronic tumor hypoxia alters the phenotype of neuroblastoma cells and that NESP55 provides an excellent marker to study this phenomenon. Based on previous studies on *IGF2* expression, we have suggested the term “chromaffin metaplasia” for the change in phenotype [12], and our present findings are summarized schematically in Figure 9. The data imply existence of simultaneous processes in stroma-rich tumors enabling maturation either towards a chromaffin or a ganglionic phenotype in neighbouring cells, putatively reflecting alternative embryonal programmes in autonomic nervous system development.

Although the chromaffin tumor phenotype in neuroblastoma is easily revealed by analyzing expression of NESP55 and *IGF2*, the corresponding morphological features are relatively inconspicuous. This is in line with the small cell size and subtle nuclear characteristics of the SIF cell phenotype, which means that it would be easily overlooked in pathological examination of neuroblastoma. It appears that these subtle morphological signs of tumor cell maturation on routine staining previously tended to be viewed as early signs of ganglionic differentiation (e.g. see Fig. 1g-i in [6]). The current data implies instead that these morphological features observed in neuroblastoma pathology represent a chromaffin phenotype. Significantly, the current data imply that the chromaffin pathway is the main form of cell maturation in stroma-poor tumors and implies co-existence of two differentiation pathways in tumors with a stroma-rich histology. We consider that this is likely to be a significant insight into understanding differentiation in neuroblastomas. However we are not suggesting that this should alter clinical pathology criteria that are in current use, since the clinical implications (if any) of the very common process we observe in neuroblastoma are as yet unclear.

### Hypoxia-induced chromaffin metaplasia applies across the clinical spectrum

An important aspect of our findings is that hypoxia-mediated chromaffin metaplasia appears to occur across the clinical spectrum of sympathetic neuroblastic tumors, ranging from ganglioneuroma through to highly aggressive variants of the disease - and that this is also observed in cell lines. This contrasts with the recently expressed view that hypoxia induces chromaffin features only in low-risk tumors, but that in high-risk disease and in cell lines tumor hypoxia leads to a dedifferentiated tumor state, similar to that of neural crest cells (reviewed in [7]). This model is not supported by the present findings since our data is derived mainly from high-risk tumors and *MYCN* amplified cell lines, and shows clear evidence of chromaffin features in these tumors in association with hypoxia. The dedifferentiated hypoxic state reported by others has been inferred from studying signaling pathways, but we would caution that the specificity of these signals as neural crest markers is uncertain, since the cell population in question is not readily available for study in the human context. In considering why these studies have reached rather different conclusions from our own it should also be noted that we have used anoxia rather than 1–0.5% oxygen in our cell culture experiments. Importantly, the similarity between the effects of anoxia in cell culture and our observations in model tumors and clinical specimens imply that our approach is relevant to the *in vivo* setting. Interestingly, amongst *NOTCH1*, *ID2*, and *HES1* which were reported to be induced by 1–0.5% oxygen and proposed as mediators of dedifferentiation [40], [47] the two latter genes were not induced under the conditions we used. Although our observations differ, and we cannot explain this, the conclusions of the studies – that hypoxia can drive aspects of differentiation and dedifferentiation should not be regarded as necessarily contradictory because it is plausible that cancer cells simultaneously develop some features associated with both differentiation and de-differentiation. Although cultured cell lines exposed to hypoxia/anoxia provide a useful model system this approach will only recreate some aspects of the complex situation in human tumors. Our findings are based primarily on clinical specimens, and it is not surprising that *in vitro* studies using clonal cell lines only capture some aspects. A specific example is that in clinical specimens (and xenografts of one cell line) we observed a correlation between hypoxia and chromograinin A expression but exposure to cell lines to anoxia in vitro did not result in increased expression.

### NESP55 in neuroblastoma - concluding remarks and future prospects

We conclude that expression of NESP55 is a sensitive marker for hypoxia-dependent chromaffin metaplasia in neuroblastoma, which represents the dominant form of differentiation in this group of tumors. Determining the mechanistic link(s) between hypoxia and chromaffin differentiation will require further studies. The role of HIF2α should be of particular interest in view of its strong expression in hypoxic neuroblastoma cells relative to HIF1α and its expression in chromaffin cell types.

The high specificity and sensitivity of NESP55 for an early chromaffin phenotype in fetal tissues makes it a valuable complement to other markers of the sympathetic subphenotype. Its hypoxia-dependent expression in neuroblastoma may reflect an important role for hypoxia in the normal development and stress-adaptation of the fetus. The clinical and therapeutic significance of such hypoxia-induced chromaffin metaplasia clearly merits further study, both in terms of embryonal/fetal adaptation to limited oxygen and in terms of neuroblastoma biology. We envisage that incorporation of this marker in the diagnostic panel for neuroblastoma will provide an important tool for improved diagnostic accuracy and for improved biological understanding of this complex set of tumors.
